# Falls Among High- and Low- Frequency Sleep Medication Users With and Without Dementia

**DOI:** 10.1093/geroni/igab046.746

**Published:** 2021-12-17

**Authors:** Loretta Anderson, Alexandra Wennberg

**Affiliations:** 1 University of Maryland, Baltimore, School of Medicine, Reisterstown, Maryland, United States; 2 Karolinska Institutet, Stockholm, Stockholms Lan, Sweden

## Abstract

Difficulty with sleep and falls are prevalent among older adults. Sleep medication use is associated with falls in older adults, but little is known about its impact in older adults with dementia. We used data from the 2011 National Health and Aging Trends Study to assess the association of low- versus high- frequency sleep medication use with falls in older adults with self-reported dementia. In our fully adjusted model, among those with dementia, high-frequency sleep medication users were more likely to fall than low-frequency sleep medication users (OR=3.86, 95% CI: 1.31, 11.37). Among those without dementia, high-frequency sleep medication users were more likely to fall than low-frequency sleep medication users (OR=1.40, 95% CI: 1.11, 1.77). Reducing sleep medication use in older adults with and without dementia may help reduce the risk of falls and fall-related outcomes in older adults.

